# A Rapid, Efficient Method for Anodic Aluminum Oxide Membrane Room-Temperature Multi-Detachment from Commercial 1050 Aluminum Alloy

**DOI:** 10.3390/nano14141216

**Published:** 2024-07-17

**Authors:** Chin-An Ku, Chia-Wei Hung, Chen-Kuei Chung

**Affiliations:** Department of Mechanical Engineering, National Cheng Kung University, Tainan 701, Taiwan

**Keywords:** anodic aluminum oxide, 1050 aluminum alloy, membrane detachment, one-step anodization process, repetition

## Abstract

For commercial processes, through-hole AAO membranes are fabricated from high-purity aluminum by chemical etching. However, this method has the disadvantages of using heavy-metal solutions, creating large amounts of material waste, and leading to an irregular pore structure. Through-hole porous alumina membrane fabrication has been widely investigated due to applications in filters, nanomaterial synthesis, and surface-enhanced Raman scattering. There are several means to obtain freestanding through-hole AAO membranes, but a fast, low-cost, and repetitive process to create complete, high-quality membranes has not yet been established. Here, we propose a rapid and efficient method for the multi-detachment of an AAO membrane at room temperature by integrating the one-time potentiostatic (OTP) method and two-step electrochemical polishing. Economical commercial AA1050 was used instead of traditional high-cost high-purity aluminum for AAO membrane fabrication at 25 °C. The OTP method, which is a single-step process, was applied to achieve a high-quality membrane with unimodal pore distribution and diameters between 35 and 40 nm, maintaining a high consistency over five repetitions. To repeatedly detach the AAO membrane, two-step electrochemical polishing was developed to minimize damage on the AA1050 substrate caused by membrane separation. The mechanism for creating AAO membranes using the OTP method can be divided into three major components, including the Joule heating effect, the dissolution of the barrier layer, and stress effects. The stress is attributed to two factors: bubble formation and the difference in the coefficient of thermal expansion between the AAO membrane and the Al substrate. This highly efficient AAO membrane detachment method will facilitate the rapid production and applications of AAO films.

## 1. Introduction

Anodic aluminum oxide (AAO) is widely used in science and industry. However, the current fabrication methods are still based on a traditional low-temperature and two-step process from high-purity aluminum (>99.99%) [1,2,3,4,5]. In order to reduce costs and achieve the high-efficiency production of AAO, it is necessary to increase the reaction temperature, the voltage, or apply a one-step process. Various applications of AAO have been developed after researching this topic for several decades, such as nanomaterial synthesis [6,7,8], humidity sensors [9,10,11], bio-applications [12,13,14,15], and surface property modification [16,17]. Moreover, the detachment of the AAO membrane for further applications has become an important research direction. Freestanding AAO films can also be used in many fields, such as for the synthesis of nanowires [18], membrane filters [19,20,21,22], and surface-enhanced Raman scattering (SERS) applications [23,24].

There are several methods of obtaining through-hole AAO films: for instance, the chemical method [5,25,26,27,28], the voltage reduction method [29], the reverse-bias voltage method [18,30,31,32], the pulse voltage method [33,34,35], and the two-layer anodization method [36,37]. In the first chemical method, it is inevitable to use solutions containing Cu^2+^ or Hg^2+^ [26,27,28], which are harmful to the environment. Also, it inevitably causes material waste, and the commercial AAO membranes fabricated following this method have the disadvantage of an irregular pore structure. Therefore, chemical etching with a heavy-metal solution has been avoided in recent experiments. The reverse-bias voltage method is the most common method used in papers. The mechanism of AAO detachment relies on the generation of H_2_ at the Al/AAO interface. Although a complete AAO film can be obtained, the detachment process takes about 13 to 20 min to finish, which is relatively time-consuming. Additionally, the bias voltage has to be precisely controlled, and the AAO thickness must exceed a certain degree, 60 μm, to avoid membrane cracks. On the other hand, the two-layer anodization method proposed by Masuda et al. [36] can achieve a complete AAO membrane several times by etching a sacrificial layer grown in concentrated sulfuric acid. However, the anodization process takes more time, around 13 h, because of the growth of the third anodization layer, and the electrolyte used may also affect the environment. To improve the fabrication process, Zhang et al. proposed to grow the sacrificial layer by annealing, resulting in a serious pore-widening phenomenon [37]. The pulse voltage detachment method is the most efficient for AAO membrane peeling; it is a fast, one-step, multi-pulse process, but it does not demonstrate membrane integrity and the possibility of repeating this process for multiple membranes, which reduce the membrane’s quality and increase material waste [33,34,35,38]. Generally, the HClO_4_-C_2_H_5_OH solution used for the detachment process is the same as that used in the electropolishing pre-treatment, which can avoid the waste of the solution; a voltage 15 V higher than the anodization voltage was recently suggested for the voltage applied during the detachment process [35,38,39]. In early 2006, Xia et al. [34] applied 50 V, which is 10 V higher than the anodization voltage for 3 s, to separate the AAO membrane, but the through-hole structure had an uneven, small pore diameter, together with a residual barrier layer. In 2002, pulse voltage detachment 15 V higher than the anodization voltage was proposed by Paterson et al. [38], establishing the foundation for subsequent research efforts. An even higher voltage, 15 V higher, was proposed by Sulka et al. [35] to detach the AAO membrane, using 55 V for 3 s, from 1 to 10 pulses, and a low voltage of 0.3 V for 3 s, following the main voltage of 55 V. In 2019, Sulka et al. [39] contributed to the literature by publishing photos of the AAO membrane obtained by the pulse voltage detachment method, but the corners were incomplete, and the process was still not repeatable. In brief, all the research mentioned above used high-purity aluminum (>99.99%) for the AAO membrane detachment process, with the disadvantages of a long process time [18,31,32,36,37], no complete membrane photos, and non-repeatable processes [33,34,35,38], alongside other restrictions. Therefore, establishing a rapid process to obtain consistently uniform nanostructured AAO membranes is a worthwhile research direction.

In this paper, we propose a one-step anodization in 0.3 M oxalic acid at 25 °C to produce AAO from economical low-purity AA1050 and a novel one-time potentiostatic (OTP) method in order to obtain multi-detached AAO complete membranes over a short process time. The OTP method consisted of applying direct current anodization (DCA) at 40 V to fabricate AAO and detaching the membrane at 50 V. Furthermore, we achieved repetition and acquired a complete film by applying the two-step electropolishing method to solve the difficulty of the AAO being easily broken. The mechanism of the OTP method is also discussed by its three major factors, including the Joule heating effect, the dissolution of the barrier layer, and stress effects. The current was concentrated on the solution barrier layer due to its lower resistance compared to the wall barrier, and the increasing Joule heating led to the chemical dissolution of the barrier. In addition, the stress generated during the AAO membrane detachment process played an important role for membrane integrity. The stress was attributed to two factors: bubble formation and the difference in the coefficient of thermal expansion at the AAO/Al interface, which cause upright stress and lead to membrane cracking. Therefore, controlling the stress in an allowable range during the membrane peeling process is the measure of a successful process. In terms of membranes with a nanostructure, the potential applications as filters and sensors are closely related to the nanostructure of the AAO membrane, so the pore size distribution is hereby verified to examine the consistency of the membrane.

## 2. Materials and Methods

**Figure 1:**
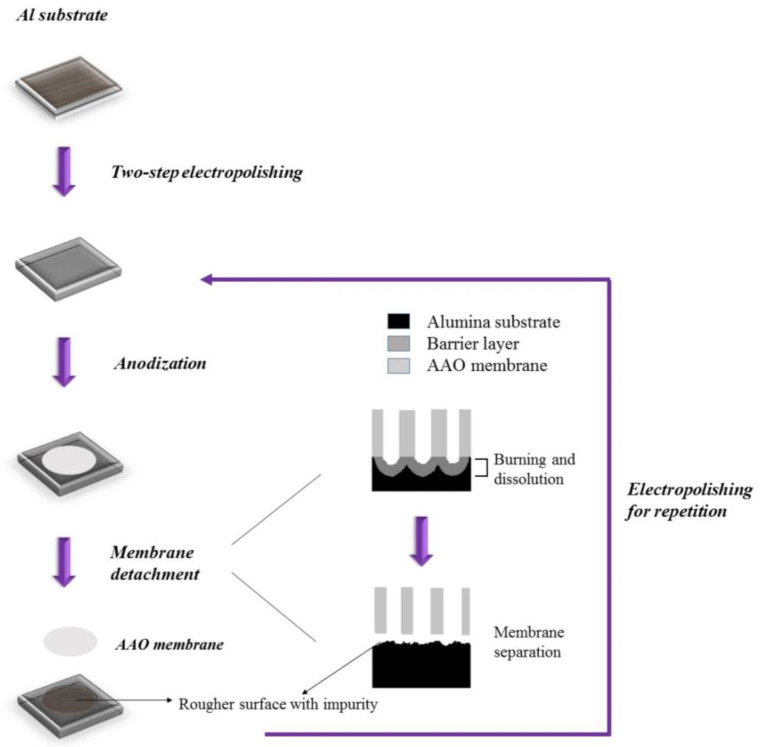
The experimental process flow diagram.

The experimental process flow is shown in Figure 1. The commercial 1050 aluminum alloy was cut into pieces 2.5 cm × 2.5 cm in size and then electropolished by a two-step process. The first step of coarse polishing was performed at 20 V in a mixture of HClO_4_:C_2_H_5_OH = 1:1 (*v*/*v*) for 1 min at 0 °C, and the second step of polishing was conducted in a HClO_4_:C_2_H_5_OH = 1:4 (*v*/*v*) solution, under the same conditions, for 5 min [40,41]. The one-step anodization experiments were performed in 0.3 M oxalic acid by DCA at 40 V, 25 °C, for 3 h. The formation of the AAO was achieved using a potentiostat (Jiehan 5000, Taichung, Taiwan) and a three-electrode electrochemical system with platinum mesh as the counter electrode, the specimen as the working electrode, and calomel as the reference electrode. After anodization, the HClO_4_:C_2_H_5_OH = 1:1 (*v*/*v*) solution was used as the electrolyte for the AAO detachment process. We applied DC 50~55 V at 25 °C for 3 s and 20 s with the platinum mesh as the counter electrode and the specimen as the working electrode. For the repetition, the surface for multi-anodization was polished to reduce surface roughness by prolonging the two-step polishing with HClO_4_:C_2_H_5_OH = 1:1 (*v*/*v*) for 2 min and HClO_4_:C_2_H_5_OH = 1:4 (*v*/*v*) for 10 min, under the same condition. The detached AAO membrane was coated with 10 nm Pt for scanning electron microscope (SEM) observation.

The morphology and pore characteristics of the AAO membranes were observed with a high-resolution field emission scanning electron microscope (HR-FESEM, HITACHI, SU-5000, Tokyo, Japan). In order to illustrate the pore distributions in detail, SEM micrographs and the AAO membranes were further analyzed with commercial software (ImageJ ver. 1.53t).

## 3. Results

**Figure 2:**
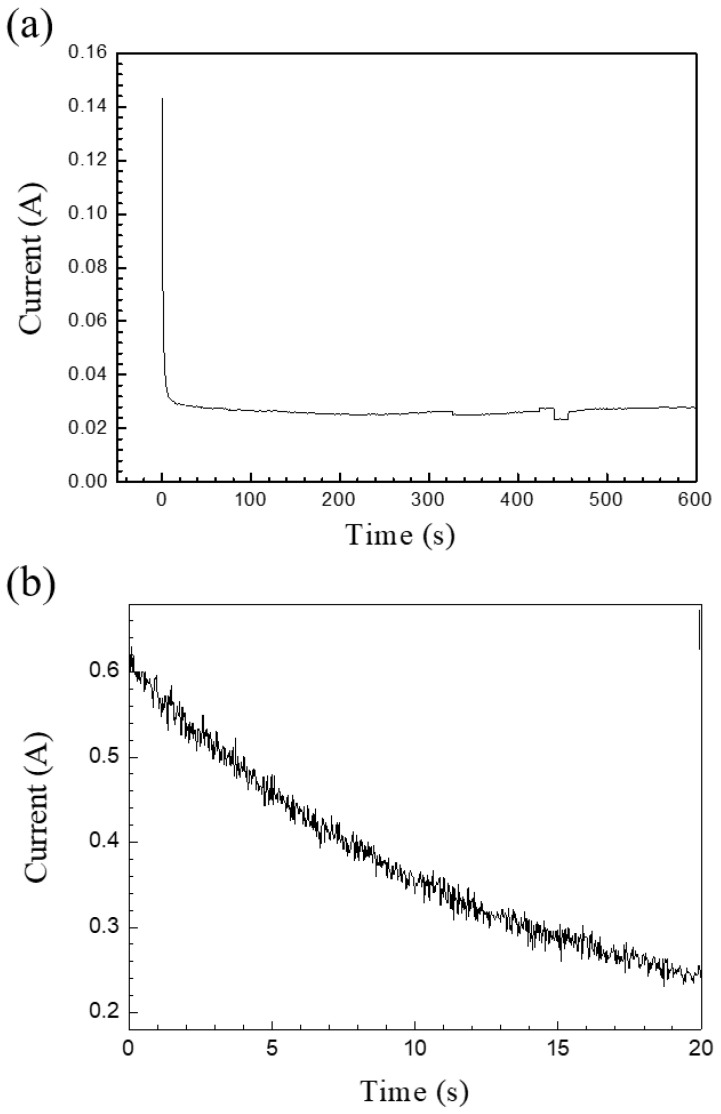
(**a**) The current–time diagram of AAO anodized by the potentiostatic method at 40 V in 0.3 M oxalic acid for 3 h at 25 °C. (**b**) The I-t characteristic curve during the detachment of AAO from the Al substrate.

Figure 2a shows the current–time diagram for the first 10 min of the one-step anodization process. The specimen was anodized using a potentiostatic method at 40 V in 0.3 M oxalic acid for 3 h at 25 °C. Initially, the direct reaction on the aluminum alloy resulted in a high current; then, it decreased sharply in a short period due to barrier oxide formation. After a few seconds, the current stabilized and remained constant for the remaining 3 h. Figure 2b illustrates the current–time diagram of the AAO detachment process in a solution of HClO_4_ and C_2_H_5_OH in a 1:1 ratio at 50 V for 20 s. It reveals that the current was initially quite high, which promoted the dissolution of the barrier layer between the AAO and the aluminum substrate. As the barrier layer dissolved, the current decreased, leading to the separation of the aluminum oxide film.

**Figure 3:**
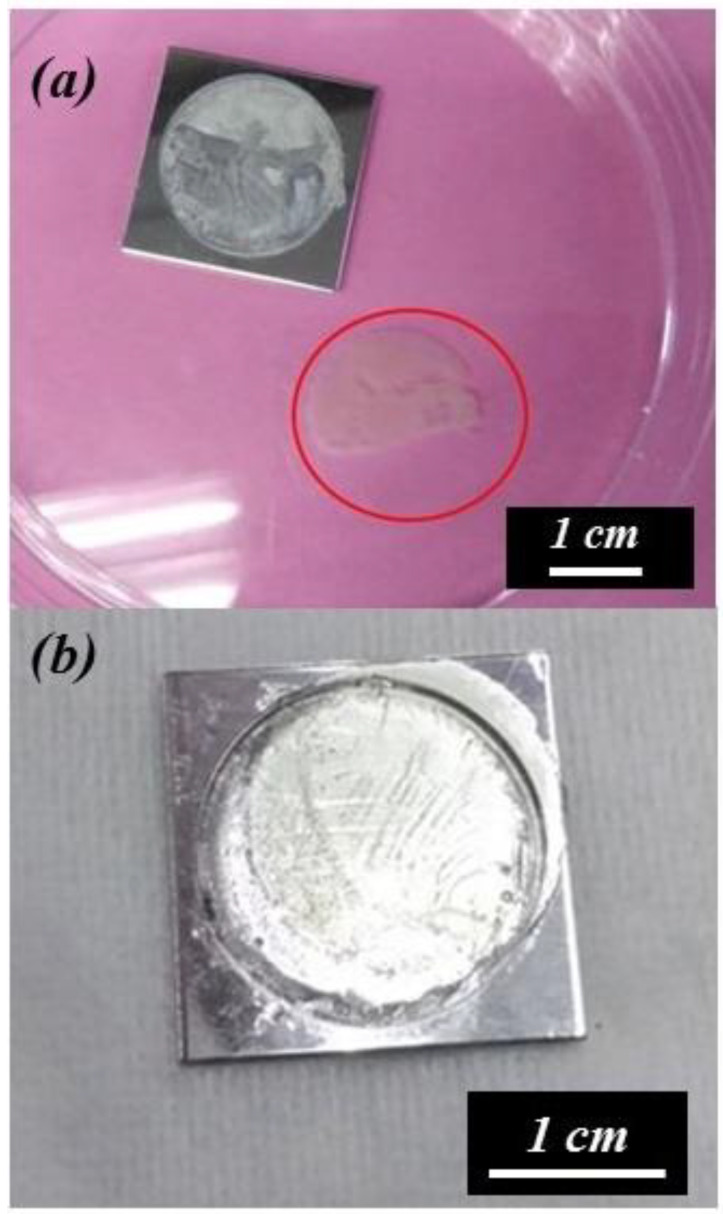
The optical photograph of the detached AAO membrane and the substrate. Potentiostatic detachment method performed under (**a**) 55 V for 3 s, leading to membrane cracking, and (**b**) 50 V for 20 s, resulting in a complete membrane.

Figure 3a,b are optical photographs of the AAO and Al substrate detached for the first time at OTPs of 55 V for 3 s and 50 V for 20 s, respectively. The membrane obtained at 55 V for 3 s is detached but appears broken, likely due to uneven peeling stress. In contrast, the membrane detached at 50 V for 20 s forms a complete, crack-free circle with a diameter of 2 cm. By optimizing the voltage from 55 V to 50 V and extending the detachment time from 3 s to 20 s, the stress effect can be controlled within an acceptable range, resulting in intact membranes. The film is completely separated without defects or cracks thanks to the lower reaction rate and fewer bubbles. The diameter of the AAO film is 2 cm, corresponding to a circular area of about 3.14 cm^2^, which matches the working area of our holder.

**Figure 4:**
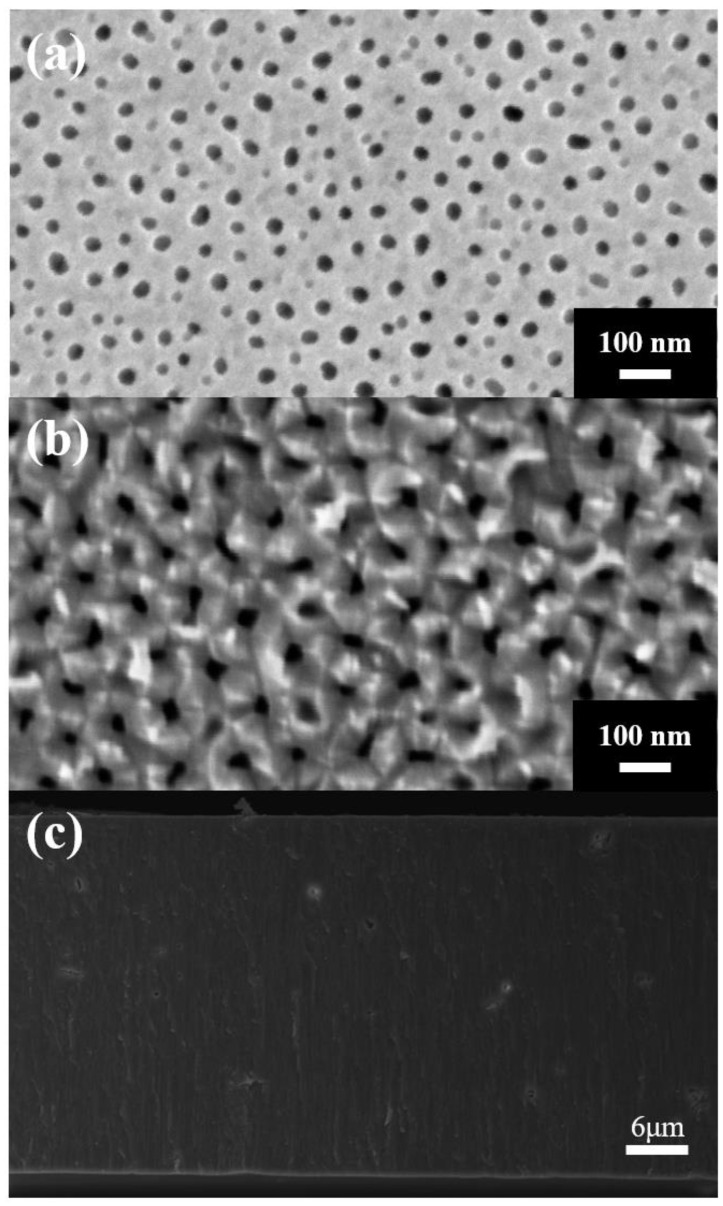
The SEM micrograph of the AAO membrane from (**a**) top, (**b**) bottom, and (**c**) cross-section views.

Figure 4 shows the SEM micrographs of the AAO membrane from the top (a), bottom (b), and cross-section (c). The ImageJ analysis revealed that the pore diameter (D_p_) and interpore distance (D_int_) of the nanoporous AAO were 38 nm and 94 nm, respectively, in the top view (Figure 4a), and 39 nm and 96 nm in the bottom view (Figure 4b). These data demonstrate the consistency of the pore structure on both sides of the freestanding film. The AAO thickness was 37.8 μm (Figure 4c), with a growth rate of approximately 12.6 μm/h. This growth rate was faster than that achieved with traditional mild anodization, indicating that increasing the anodization temperature enhances efficiency. 

**Figure 5:**
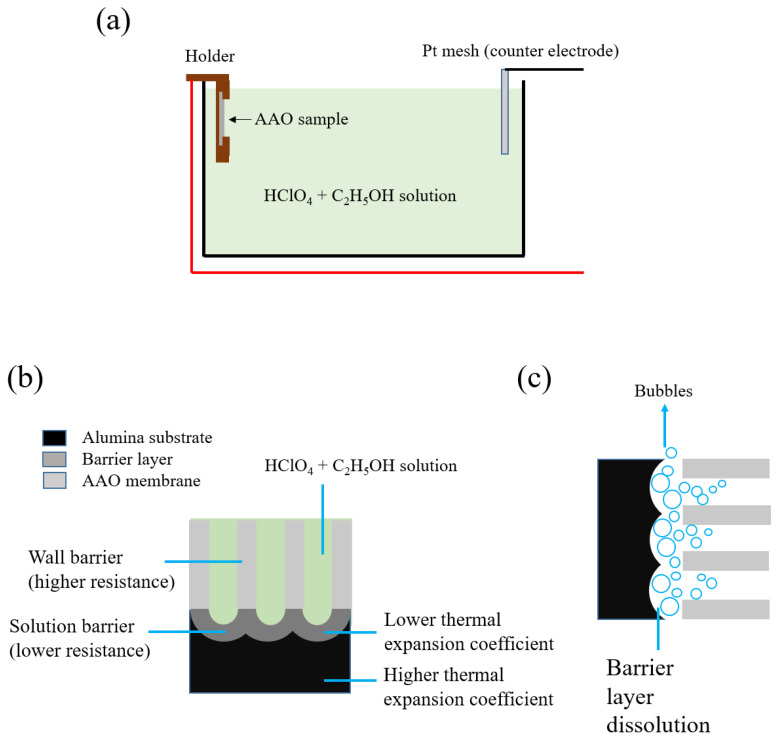
The AAO membranes’ detachment mechanism. (**a**) The electrochemical cell setup of the AAO membrane detachment process. (**b**) The schematic diagram of the AAO/Al interface during the detachment process. (**c**) The schematic diagram of bubble generation during the membrane detachment process.

The AAO membrane detachment mechanism of our OTP method is explained in Figure 5. Figure 5a shows the electrochemical cell setup for the AAO membrane detachment process, while Figure 5b,c present schematic diagrams of the AAO/Al interface and bubble generation during membrane detachment. The main factors contributing to membrane detachment are Joule heat, chemical dissolution, and peeling stress. During membrane peeling, the current concentrates through the barrier layer of the pores with the electrolyte due to the lower-resistance path compared to the higher resistance of the AAO wall barrier (Figure 5b), generating a large amount of Joule heat. This heat causes a rapid temperature increase in the barrier layer, accelerating its chemical dissolution and opening the pore bottoms.

The integrity of the AAO membranes is mainly affected by stress effects. During the detachment process, bubbles are generated by chemical dissolution and rise to the Al/AAO interface (Figure 5c). Initially, bubbles form at the lower-resistance barrier and expand towards the barrier beneath the wall, influencing membrane peeling with uneven vertical-to-membrane gas stress from bubble formation and escape. This is due to the AAO pores being upright with respect to the Al/AAO interface in the electrochemical cell (Figure 5a), which can easily lead to membrane cracks, especially in large areas with greater stress.

In our process, we applied 50 V for 20 s instead of 55 V for 3 s to detach the AAO membrane, reducing chemical dissolution and bubble formation. This controls the stress within an allowable range, resulting in a complete membrane. Another factor generating stress is the different thermal expansion coefficients of aluminum and alumina, which are 23.2 × 10^−6^/°C and 7 × 10^−6^/°C, respectively. When the temperature of the barrier layer beneath the pore and the wall increases unevenly, additional uneven thermal stress is applied to the Al/AAO interface during peeling. By reducing the voltage to 50 V and prolonging the reaction time to 20 s, we can reduce sudden stress generation and maintain an acceptable stress over a longer peeling time. This ensures membrane integrity and the repeatability of the process.

Table 1 lists the comparison of the fabrication and results of AAO membrane peeling reported in several journals to ours. In addition to commercial or traditional methods of Cu^2+^ or Hg^2+^ solutions, the methods used can be divided into three categories: the reverse-bias voltage method [5,19,25,26,27,28,29,30], the pulse voltage method [32,33,38], and the two-layer anodization method [34,35]. It is noted that only our work successfully produced through-hole AAO membranes from low-purity aluminum. Moreover, it took only 3 h to prepare the AAO, which proved that our process had the advantages of being fast and having a high efficiency and a low cost. In terms of membrane detachment, some teams claim that they took only 3 s of the pulse voltage detachment method to achieve AAO film separation, but none of these articles mention their film’s integrity and process repeatability. Although the reverse-bias voltage method and the two-layer anodization method can obtain complete membranes and repetition on the same substrate, these processes take more time, around 8~32 h, and steps. Also, the reverse-bias voltage method has film thickness restrictions and the voltage need to be precisely controlled during several stages. The two-layer anodization method inevitably uses a solution that is harmful to the environment or causes the large pore widening phenomenon. Furthermore, it is based on growing a sacrificial layer to achieve AAO membrane separation. This requires a total of three anodizations [36,37] and, possibly, an additional annealing process [37], which prolongs the time needed to obtain a single membrane. In this study, we demonstrate our method’s ability to repeatedly detach a complete AAO membrane, five times, and each detachment process took only 20 s. Overall, we proposed a fast, low-cost, and relatively green process to detach complete through-hole AAO membranes in one step, repeatedly, which can solve the disadvantages of complex steps, time-consuming methods, membrane integrity, non-repeatability, or toxic solutions, as mentioned above.

**Figure 6:**
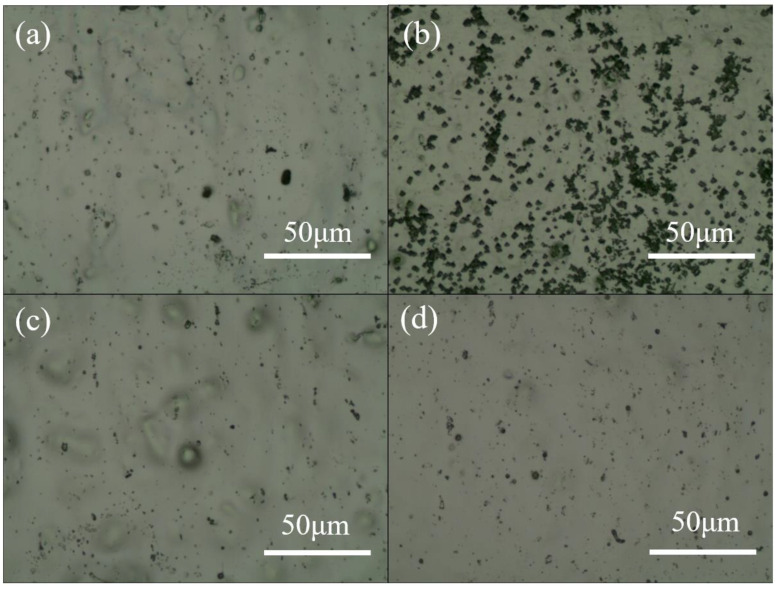
The optical micrographs of (**a**) AA1050 after two-step electropolishing; (**b**) AA1050 after first AAO membrane detachment; (**c**) two-step electropolishing on (**b**); and (**d**) prolonged two-step electropolishing on (**b**).

Figure 6 shows the optical microscope images from the aluminum substrate surface during the experimental process: (a) after the initial two-step electrochemical polishing; (b) after AAO membrane detachment; (c) after performing the same polishing parameters on the substrate following AAO membrane detachment; and (d) after further extending the polishing time for HClO_4_:C_2_H_5_OH= 1:1 by 2 min and for HClO_4_:C_2_H_5_OH = 1:4 by 10 min. During the AAO membrane detachment process, a significant amount of Joule heat is generated, which makes the aluminum substrate surface become uneven, as shown in (b). This is also the main reason why the pulsed voltage detachment method with similar reaction mechanisms cannot be repeated. Therefore, we developed and improved a two-step electrochemical polishing method to overcome defects and roughness on the substrate, enabling the repetition of AAO membrane creation on the same substrate. However, since the surface of the aluminum substrate becomes rougher after AAO membrane detachment compared to its initial state, it is observed that there are still some rough traces under the same polishing conditions in (c). When we extended the polishing time for HClO_4_:C_2_H_5_OH= 1:1 by 2 min and for HClO_4_:C_2_H_5_OH = 1:4 by 10 min, as shown in (d), the defects were significantly reduced, allowing the subsequent repeated detachment process to proceed smoothly.

**Figure 7:**
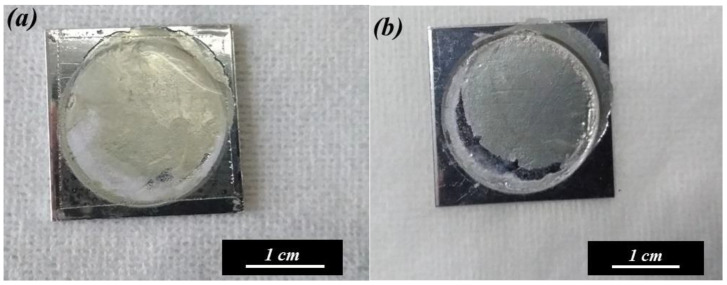
The photographs of the AAO membrane after the (**a**) third and (**b**) fifth repetition from the AA1050 substrate.

**Figure 8:**
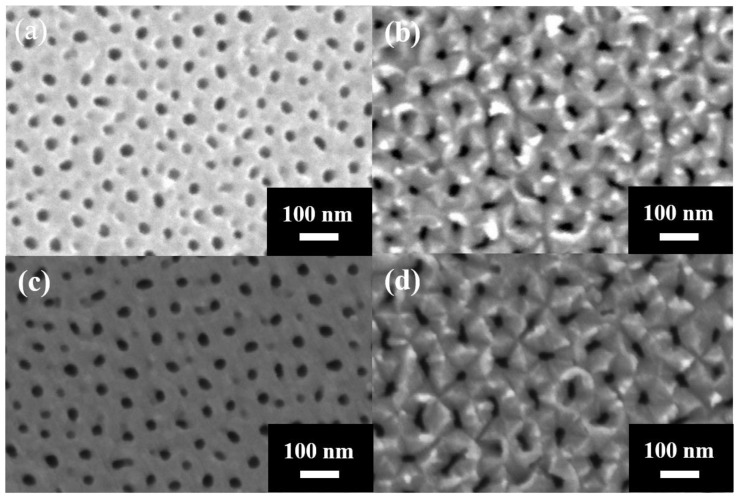
The SEM micrographs of the AAO surface from the top (**a**,**c**) and bottom (**b**,**d**) side obtained by the third (**a**,**b**) and fifth (**c**,**d**) repetitions of AAO membrane detachment, respectively.

Figure 7a,b show the optical images of the AAO membrane after the third and fifth detachment process, respectively. The freestanding AAO films are still quite complete after several detachment experiments, which is attributed to the multi-step electrochemical polishing. The anodization and detachment process by the potentiostatic method is a feasible technique and can be repeatedly performed to obtain stable results on the same substrate. Figure 8 shows the SEM micrographs of AAO morphology from the top side (a,c) and the bottom side (b,d). The graphs from (a,b) and (c,d) are the AAO films after the third and fifth detachment, respectively. Following the ImageJ analysis, the average pore diameters of the membranes are 39 nm, 39 nm, 38 nm, and 40 nm, corresponding to Figure 8a–d, respectively. After repeated experiments, the obtained AAO films were still highly consistent, with a similar morphology. 

**Figure 9:**
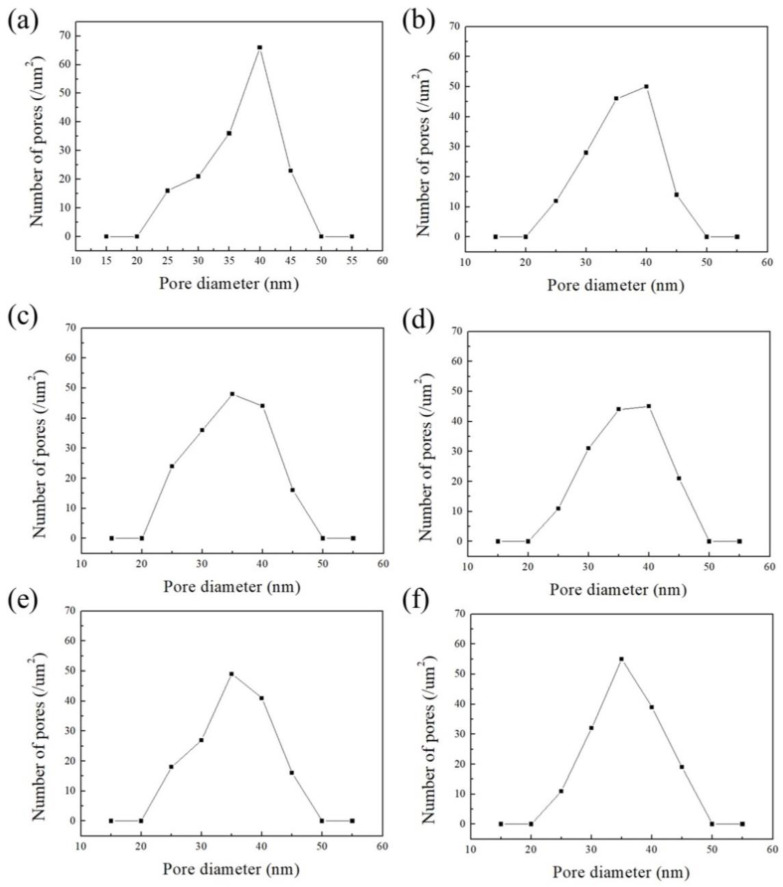
The AAO pore distribution analysis of (**a**,**b**) first detachment, (**c**,**d**) third, and (**e**,**f**) fifth repetition processes from SEM images of the AAO membrane’s (**a**,**c**,**e**) top and (**b**,**d**,**f**) bottom view.

Figure 9a,b show the AAO pore distribution analysis from the first detachment from the top and bottom view, referring to Figure 4a,b, and Figure 9c–f are from the third and fifth multi-detachment SEM images, corresponding to Figure 8a–d, respectively. The pore diameter of the AAO membrane presents a unimodal distribution, and the peaks fall between 35 and 40 nm on both sides of the membrane. In Figure 9a, the range of 38 ± 5 nm pores occupies the main distribution, and the distribution uniformity is 63%, calculated by the ratio of pores located at these main peaks to the whole amount of pores. In Figure 9b,c,e, the distribution uniformities are 63%, 59%, and 60% in the range of 38 ± 5 nm, respectively. In Figure 9d,f, the distribution uniformities are 59% and 60% in the range of 38 ± 5 nm and 40 ± 5 nm. The pore distribution is still highly consistent after five membrane detachments, which indicates that the microstructure of AAO has not changed within the multi-detachment process. It has been proven that our method can obtain high-quality AAO membranes quickly and repeatedly.

## 4. Conclusions

We propose a rapid and efficient method for the multi-detachment of the AAO membrane at room temperature by integrating the OTP method and two-step electrochemical polishing. This method allows for repeatedly detaching complete AAO membranes from the same substrate, overcoming the drawbacks of complex steps and time-consuming methods such as the voltage reduction method, the reverse-bias voltage method, or the two-layer anodization method, or the inability to obtain multiple complete films, such as the pulse voltage methods. Our method takes less than 200 min to fabricate a high-quality AAO membrane, and the membrane detachment process is just 20 s long. The anodization and detachment processes are conducted at 25 °C, which overcomes the shortcomings of low-temperature reactions in the past and improves the manufacturing efficiency of AAO through-hole membranes. We repeated the detachment of the AAO membranes five times on the same substrate, and the pore diameters were highly consistent, between 35 and 40 nm, with a unimodal distribution, according to our SEM micrograph analysis. The improved two-step electrochemical polishing was applied to reduce the surface roughness and impurities of the substrate, key to successfully separating AAO films multiple times from the same substrate. Furthermore, we explained the AAO membrane peeling mechanism from multiple aspects, including Joule heating, chemical dissolution, and stress effects. The stress was attributed to bubble formation and the difference in the coefficient of thermal expansion between the AAO membrane and the Al substrate. Reducing the detachment voltage from 55 V to 50 V and prolonging the process time from 3 s to 20 s can control the stress effect within an allowable range. These are the main factors to consider in order to obtain a complete membrane using our OTP method.

## Figures and Tables

**Table 1:** Comparison of AAO membrane detachment methods and results.

Ref.	Method	Al Purity	Anodization Step/Solution	AAO Fabrication Time (h)	Detachment Time	Photographs of Complete Membrane	Repetition of AAO Membrane
[22] (Commrcial membrane)	Chemical etching method	99.999%	NA	NA	NA	Complete	NA
[18]	Reverse-bias voltage method	99.999%	two-step/Sulfuric acid	25	20 min	Complete	6 times
[31]	Reverse-bias voltage method	99.999%	two-step/ Sulfuric acid	44	13 min	Local *	NA
[32]	Reverse-bias voltage method	99.999%	two-step/oxalic acid	8–32	30–90 s	Local *	5 times
[33]	Pulse voltage method	99.999%	two-step/oxalic acid	28	3 s (1 cycle)	Local *	NA
[34]	Pulse voltage method	99.99%	two-step/oxalic acid	5	3 s (1 cycle)	Local *	NA
[35]	Pulse voltage method	99.999%	two-step/oxalic acid	5	3–60 s (1–10 cycle)	Local *	NA
[39]	Pulse voltage method	99.999%	two-step/sulfuric acid	12–20	3–60 s (1–10 cycle)	Partial	NA
[36]	Two-layer anodization method	99.999%	three-step/sulfuric acid	13.5	15 min	Complete	10 times
[37]	Two-layer anodization method	99.999%	three-step/oxalic acid	27	75 min	Complete	4 times
Ours	Short one-time potentiostatic method	Al 1050 alloy (~99.5%)	one-step/oxalic acid	3	20 s	Complete	5 times

* SEM images only.

## Data Availability

Data are presented in the coauthors’ research results, and the schematic drawing is available upon request.
